# Treatment with Exogenous Trypsin Expands In Vitro Cellular Tropism of the Avian Coronavirus Infectious Bronchitis Virus

**DOI:** 10.3390/v12101102

**Published:** 2020-09-29

**Authors:** Phoebe Stevenson-Leggett, Sarah Keep, Erica Bickerton

**Affiliations:** The Pirbright Institute, Ash Road, Woking, Surrey GU24 0NF, UK; phoebe.stevenson-leggett@pirbright.ac.uk (P.S.-L.); sarah.keep@pirbright.ac.uk (S.K.)

**Keywords:** coronavirus, animal coronavirus, cleavage, tropism, glycoprotein, spike protein

## Abstract

The *Gammacoronavirus* infectious bronchitis virus (IBV) causes a highly contagious and economically important respiratory disease in poultry. In the laboratory, most IBV strains are restricted to replication in ex vivo organ cultures or in ovo and do not replicate in cell culture, making the study of their basic virology difficult. Entry of IBV into cells is facilitated by the large glycoprotein on the surface of the virion, the spike (S) protein, comprised of S1 and S2 subunits. Previous research showed that the S2′ cleavage site is responsible for the extended tropism of the IBV Beaudette strain. This study aims to investigate whether protease treatment can extend the tropism of other IBV strains. Here we demonstrate that the addition of exogenous trypsin during IBV propagation in cell culture results in significantly increased viral titres. Using a panel of IBV strains, exhibiting varied tropisms, the effects of spike cleavage on entry and replication were assessed by serial passage cell culture in the presence of trypsin. Replication could be maintained over serial passages, indicating that the addition of exogenous protease is sufficient to overcome the barrier to infection. Mutations were identified in both S1 and S2 subunits following serial passage in cell culture. This work provides a proof of concept that exogenous proteases can remove the barrier to IBV replication in otherwise non-permissive cells, providing a platform for further study of elusive field strains and enabling sustainable vaccine production in vitro.

## 1. Introduction

Infectious bronchitis virus (IBV) is a member of the *Orthocoronavirinae*. There are four genera, denoted *Alphacoronavirus*, *Betacoronavirus*, *Gammacoronavirus* and *Deltacoronavirus*, with members of each known to infect a range of hosts causing a variety of diseases in both humans and animals. The most (in)famous members of the family are the betacoronaviruses severe acute respiratory syndrome coronavirus (SARS-CoV) [1,2], Middle East respiratory syndrome coronavirus (MERS-CoV) [3] and the recently emerged SARS-CoV-2 [4], all of which can cause severe disease in humans. IBV, a *Gammacoronavirus*, infects domestic fowl and causes infectious bronchitis (IB), a highly contagious respiratory disease that results in poor weight gain and reduced egg production and is therefore of significant economic importance to poultry industries worldwide. IBV is perhaps unusual in comparison to some of the other more well-known coronaviruses as there are many co-circulating strains of different serotypes and genotypes that inflict varying degrees of clinical disease [5,6,7]. 

Effective vaccination against IB is an ongoing challenge due to the many current co-circulating and constantly emerging genotypes and serotypes, with current vaccines offering limited cross protection [8,9]. There is therefore a drive to both rationally attenuate IBV through specific modifications to the IBV genome [10] and to reduce the dependency on the supply of embryonated hens’ eggs for vaccine production. Both issues are compounded as many strains of IBV, including economically important strains such as 4/91(UK), exhibit restricted cell tropism and cannot be propagated in cell culture [7,11]. Not only does this restrict the possibility of vaccine production in cell culture, it also limits the study of the molecular biology of IBV replication to strains such as the attenuated laboratory adapted strain, Beaudette [12]. The inability to study a wide variety of strains in vitro hinders IBV research, vaccine development and ultimately impacts rapid response to newly emerging field strains.

Coronavirus tropism is determined by the spike (S) glycoprotein, a large type I transmembrane type 1 fusion protein that protrudes outwards from the virion surface [13,14]. The S glycoprotein is responsible for both attachment to host cells and the fusion of viral and cellular membranes, ultimately leading to virus entry, genome release and replication. In many coronaviruses, including murine hepatitis virus (MHV), transmissible gastroenteritis virus (TEGV) and feline infectious peritonitis (FIPV), the S glycoprotein has been linked to tropism both in vitro and in vivo [13,15,16,17]. The S glycoprotein also has a role in zoonotic transmission, highlighted by SARS-CoV, in which two mutations within the S glycoprotein enabled the zoonotic transfer from palm civets into humans [18]. For IBV, the role of the S protein in cellular tropism has been investigated using recombinant viruses, where the S protein was shown to confer the cellular tropism of the strain from which it was derived [11,19,20]. Furthermore, the extended in vitro tropism of the recombinant IBV (rIBV) Beau-R, a molecular clone of the pathogenic Beaudette-CK strain, has been associated with the S glycoprotein [21]. Beau-R is notably and unusually able to replicate in the continuous cell lines Vero, Baby Hamster Kidney (BHK) and the chicken fibroblast DF-1 cell line, as well as primary chicken kidney (CK) cells [22,23].

The structure of the coronavirus S glycoprotein is complex, with three individual protomers assembling to form one trimeric S glycoprotein [14,24,25]. Each S glycoprotein comprises two subunits; S1, the globular head of the protein containing the receptor binding domain, and S2, the stalk segment that contains the fusion peptide (FP) and tethers the glycoprotein to the virion membrane [14]. The IBV S1 subunit is considered to induce the majority of virus-neutralising antibodies during in vivo infection [26,27,28]. Dramatic conformational changes are observed in the S glycoprotein during entry into host cells [29]. These changes are required for efficient exposure of the FP, and initiation of membrane fusion. In many coronaviruses, including IBV, cleavage between the S1 and S2 subunits is essential for entry, with the primary cleavage site lying at the junction between the two subunits [13,30]. Notably, this is not required for infection of MERS-CoV and SARS-CoV, which both display uncleaved S proteins, although S1/S2 cleavage is required for MERS-CoV entry into certain cell types [31]. More recently, S1/S2 cleavage has been described as essential for SARS-CoV-2 entry into human lung cells [32]. A secondary cleavage site has also been identified in the S2 subunit of some CoVs, including IBV Beaudette strains, SARS-CoV and MERS-CoV, termed the S2′ site [33,34,35]. In the betacoronaviruses SARS-CoV and MERS-CoV, the S2′ site has been demonstrated to be a crucial determinant of particular entry pathways and a key factor in viral fusion mechanisms. This site has been assigned a determinant of cellular tropism in IBV, where it has been shown to be associated with the replication of Beau-R in Vero cells [21]. In addition to the S glycoprotein, protease expression has also been linked to cell and tissue tropism in coronaviruses including the Beaudette strain of IBV [36]. Recent investigations have linked proteolytic cleavage of the spike glycoprotein to the capacity of a given coronavirus to overcome the species barrier. Research using recombinant MERS-CoVs explored this by employing exogenous trypsin as a tool to expand the tropism of the virus in vitro [37]. Furthermore, propagation of the *Alphacoronavirus* porcine epidemic diarrhoea virus (PEDV) in cell culture requires the addition of exogenous trypsin, as do many strains of influenza [38,39,40]. The addition of exogenous trypsin for these viruses has therefore provided a vital mechanism for the study of the molecular basis of viral replication and host responses in vitro. 

In the present study, we have assessed the effects of exogenous trypsin on three different IBV strains during infection, and assessed whether this can be used as an artificial tool to expand cell tropism, thereby enhancing the potential for in vitro research. We investigated three IBV strains, M41-CK, a pathogenic laboratory strain [19], 4/91(UK), a pathogenic field isolate [11] and the recombinant IBV (rIBV) Beau-R [23], an attenuated laboratory strain. Infections were assessed in Vero cells, a continuous cell line already licensed for vaccine production [41] and DF-1 cells, a cell line derived from chicken embryo fibroblasts [42]. Although Beau-R is able to replicate in both cell types, titres of Beau-R generated from infected Vero cells were increased 24 h post infection (hpi) in the presence of exogenous trypsin. In both Vero and DF-1 cells, titres of M41-CK were significantly increased after passage with exogenous trypsin, however no effect was observed during 4/91(UK) infection. The sequence of the M41-CK S gene was investigated after five passages in the presence of exogenous trypsin in Vero cells, with two coding mutations identified. 

## 2. Materials and Methods 

### 2.1. Cells and Viruses

Vero cells were obtained from the central services unit (CSU) at The Pirbright Institute and maintained in Eagle’s minimum essential medium (EMEM, Sigma-Aldrich, St. Louis, MO, USA) containing 10% foetal bovine serum (FBS) and 1% l-Glutamine (Sigma-Aldrich, St. Louis, MO, USA). Chicken kidney (CK) cells were prepared as previously described [43] from kidneys of 2–3 week old Rhode Island Red (RIR) chickens, reared at The Pirbright Institute. DF-1 cells were obtained from CSU and were maintained in Dulbecco’s minimum essential medium (DMEM) containing 10% FBS and 1% l-Glutamine (Sigma-Aldrich, St. Louis, MO, USA).

Stocks of IBV strains M41-CK (GenBank accession number MK728875.1), Beau-R (GenBank accession number AJ311317) and 4/91(UK) (GenBank accession number JN192154) were propagated in 9- or 10-day old SPF embryonated hens’ eggs. M41-CK is a pathogenic M41-derived CK adapted laboratory strain of the Massachusetts serotype [44]. Beau-R is an infectious clone of the attenuated Beaudette-CK strain [12,45,46] also belonging to the Massachusetts serotype. The strain 4/91(UK) is a pathogenic field strain of UK origin [11].

### 2.2. Infection of Vero and DF-1 Cells with IBV in the Presence of TPCK-Treated Trypsin

Six-well tissue culture plates of Vero or DF-1 cells were infected with IBV at a multiplicity of infection (MOI) of 0.1, diluted in serum free *N*,*N*-bis(2-hydroxyethyl)-2aminoethanesulphonic acid (BES) medium with added trypsin treated with L-(tosylamido-2-phenyl) ethyl chloromethyl ketone (TPCK-treated trypsin, Sigma-Aldrich, St. Louis, MO, USA) at concentrations of either 0.2, 1.0 or 2 µg/mL for Vero or 0.2, 0.5 or 1.0 µg/mL for DF-1 cells, in a total volume of 500 µL per well. BES medium alone or BES medium containing the same concentrations of trypsin as infected wells was used for mock infections. Cells were incubated at 37 °C in 5% CO_2_ for 1 h. Following the primary incubation step, the virus inoculum was removed, and cells were washed twice in phosphate-buffered saline (PBS) before the addition of 3 mL of BES medium with the appropriate concentration of trypsin (matched to the media in which the virus was diluted). Cells were incubated at 37 °C in 5% CO_2_ for 24 h, after which the supernatant was harvested. Quantities of infectious viral progeny in the supernatant were assessed by titration in CK cells. To prepare Vero cell lysates, cells were washed once in cold PBS after which 350 µL of radioimmunoprecipitation assay (RIPA) lysis buffer (ThermoFisher, Waltham, MA, USA) containing protease inhibitor cocktail (PIC) and ethylenediaminetetraacetic acid (EDTA) (ThermoFisher, Waltham, MA, USA) was added to each well. Cells were incubated on ice for 20 min with constant agitation. Cells were scraped into buffer and centrifuged at 10,000× *g*; supernatant was stored at −20 °C.

### 2.3. Analysis of IBV Replication Kinetics in the Presence of TPCK-Treated Trypsin

Confluent six-well tissue culture plates of Vero cells were infected with IBV M41-CK or rIBV Beau-R at a MOI of 0.01. Viruses were diluted in serum-free BES medium containing TPCK-treated trypsin at a concentration of 1.0 µg/mL or BES medium alone (untreated) in a total volume of 500 µL per well. Cells were incubated at 37 °C in 5% CO_2_ for 1 h. Following the primary incubation step, virus inoculum was removed and cells were washed twice in PBS before the addition of 3 mL of BES medium containing TPCK-treated trypsin at a concentration of 1.0 µg/mL or BES medium alone. Cells were incubated at 37 °C in 5% CO_2_. Supernatant was harvested at 1, 12, 24, 48 and 72 hpi and quantities of infectious viral progeny were assessed by titration in CK cells. 

### 2.4. Passaging IBV with TPCK-Treated Trypsin in Vero and CK Cells 

Confluent six-well tissue culture plates of Vero or CK cells were infected with IBV diluted at a ratio of 1:1 in serum free BES medium with added TPCK-treated trypsin (Sigma-Aldrich, St. Louis, MO, USA) at concentrations of either 0.2 or 2 µg/mL for Vero cells, or 0.2, 0.5 and 1.0 µg/mL for CK cells, in a total volume of 500 µL per well. BES medium alone or BES medium containing the same concentrations of trypsin as infected wells was used for mock wells. Cells were incubated at 37 °C in 5% CO_2_ for 1 h. Following the primary incubation step, virus inoculum was removed and cells were washed twice in PBS before the addition of 3 mL of BES medium with the appropriate concentration of trypsin (matched to the media in which the virus was diluted). Cells were incubated at 37 °C in 5% CO_2_ for 48 h, after which the supernatant was harvested. Quantities of infectious viral progeny in the supernatant were assessed by titration in CK cells. The supernatant was used for four subsequent passages in each cell type following the same protocol. Cell lysates were prepared from infected Vero cells following the removal of the supernatant. Cells were washed once in cold PBS, after which 350 µL of RIPA lysis buffer (ThermoFisher, Waltham, MA, USA) containing protease inhibitor cocktail (PIC) and EDTA (ThermoFisher, Waltham, MA, USA) was added to each well. Cells were incubated on ice for 20 min with constant agitation. Cells were scraped into buffer and centrifuged at 10,000× *g*; supernatant was stored at −20 °C.

### 2.5. Analysis of Protein Expression and S Protein Cleavage by Western Blot 

Cell lysates were thawed at room temperature and diluted 3:1 with Laemmli Sample Buffer (SB, 4X, Bio-Rad, Santa Rosa, CA, USA) containing β-mercaptoethanol (Sigma-Aldrich, MO, USA). Diluted samples were heated to 80 °C for 10 min before loading on to a Bio-Rad Protean Mini-TGX™ sodium dodecyl sulphate-polyacrylamide gel electrophoresis (SDS-PAGE) gel (4–20%). Samples were run for 1 h at 150 V alongside Bio-Rad Protein Dual Colour Standard. Proteins were transferred onto a nitrocellulose membrane following the Bio-Rad Trans-Blot^®^ turbo transfer protocol for Mini-TGX™ gels. Membranes were blocked for 1 h in PBS containing 0.1% Tween 20 and 5% Marvel milk powder. Primary antibodies were diluted in the same blocking solution and applied to membranes for 1 h at room temperature. Anti-S2 (PrioMab, ThermoFisher, Waltham, MA, USA) and anti-β-actin (Abcam, Cambridge, UK) mAbs were applied at dilutions of 1:500 and 1:1000, respectively. Membranes were washed three times in PBS containing 0.1% Tween 20 (PBS-T, Sigma-Aldrich, St. Louis, MO, USA) for 5 min. Secondary antibodies were diluted in blocking solution and applied to membranes at a dilution of 1:10,000 for 1 h at room temperature. Membranes were washed a further three times before a final wash in deionised water. Membranes were analysed using a Licor Odyssey scanner using both 700 and 800 CW channels.

### 2.6. Analysis of IBV Infection in Vero Cells by Confocal Microscopy

Vero cells were seeded on glass coverslips in 24-well tissue culture plates at 1 × 10^5^ cells/mL in EMEM (10% FBS). Cells were washed once in PBS and infected with IBV (MOI = 10), diluted in BES-containing medium. BES-containing medium was added to mock wells. Trypsin was added to half the wells at a final concentration of 1 µg/mL. Cells were incubated at 37 °C for 1 h (5% CO_2_). Cells were washed once in PBS and 1 mL of BES-containing medium with or without TPCK-treated trypsin (1 µg/mL) was added to each well. Cells were incubated at 37 °C (5% CO_2_) for 24 h. Cells were washed once in PBS, then fixed in PBS containing 4% paraformaldehyde (Electron Microscopy Services, Hatfield, PA, USA) for 20 min at room temperature. Cells were washed once in PBS then permeabilised with PBS containing 0.1% Triton X100 (Sigma-Aldrich, St. Louis, MO, USA) for 10 min at room temperature. Cells were washed once in PBS, then blocking solution was added to each well consisting of PBS containing 0.5% bovine serum albumin (BSA, Sigma-Aldrich, St. Louis, MO, USA). Cells were incubated in blocking solution for 1 h at room temperature. Blocking solution was removed and replaced with blocking solution containing primary antibodies against dsRNA (Scicons, Szirák, Hungary) and alpha tubulin (Abcam, Cambridge, UK) (both diluted 1:1000). Cells were incubated with primary antibodies for 1 h at room temperature before three 5-min incubations in PBS. Secondary antibodies (AlexaFluor488 and 568 Goat Anti-Mouse, Invitrogen, Carlsbad, CA, USA) were diluted 1:500 in blocking solution and applied to cells for 1 h at room temperature (under foil). Cells were washed three times in PBS before the addition of 4′,6-diamidino-2-phenylindole (DAPI) (Abcam, Cambridge, UK) diluted 1:10,000 in H_2_O. Cells were incubated with DAPI for 5 min before a final wash in H_2_O. Coverslips were mounted onto glass slides using VectaShield (Vector Labs, Burlingame, CA, USA) and sealed with nail varnish before analysis under a Leica confocal microscope.

### 2.7. RNA Extraction from CELL supernatant 

RNA was extracted from cell supernatant following the RNA Clean-Up protocol in the QIAGEN RNeasy Mini Kit (QIAGEN, Hilden, Germany). All steps were carried out according to the manufacturer’s instructions. RNA was eluted into 35 µL of RNAse-Free water and stored at −20 °C.

### 2.8. Reverse Transcription (RT), PCR and Sanger Sequencing 

Reverse transcription was carried out with 5 µL supernatant-derived RNA using SuperScript IV Reverse Transcriptase (Invitrogen, Carlsbad, CA, USA), according to the manufacturer’s instructions. The detection of the IBV 3′ untranslated region (UTR) was achieved using universal primers BG56 (5′-CAACAGCGCCCAAAGAAG-3′) and BG93/100 (3′-GCTCTAACTCTATACTAGCCT-5′). PCR across the S gene was carried out using primer sets covering the S1 and S2 subunits. Primers covering S1 are termed BG42 (5′-AATAATGGCAATGATGAC-3′) and BG136 (3′-AACTGCCACAAACATACTGC-5′). Those covering S2 are termed BG46 (5′-CATCAAAATCACTAATGG-3′) and BG142 (3′-AGGGATCAAATACTTCTGTG-5′). PCR products were generated using *Taq* DNA polymerase recombinant (Invitrogen, Carlsbad, CA, USA), according to the manufacturer’s instructions. An extension time of 1 min per kb was employed. PCR products were analysed by gel electrophoresis and product sizes (bp) were compared to 1 kb Plus DNA Ladder (ThermoFisher, Waltham, MA, USA). PCR products were sent to Eurofins GATC for Sanger sequencing with each primer, according to the company’s requirements. Sequencing reads were analysed using Staden software. 

### 2.9. Statistical Analyses

All statistical analyses described were performed using GraphPad Prism 8.0. Data were assessed for normality before the selection of the appropriate test. 

## 3. Results

### 3.1. Exogenous Trypsin Enhances M41-CK Replication in Vero Cells

**Figure 1:**
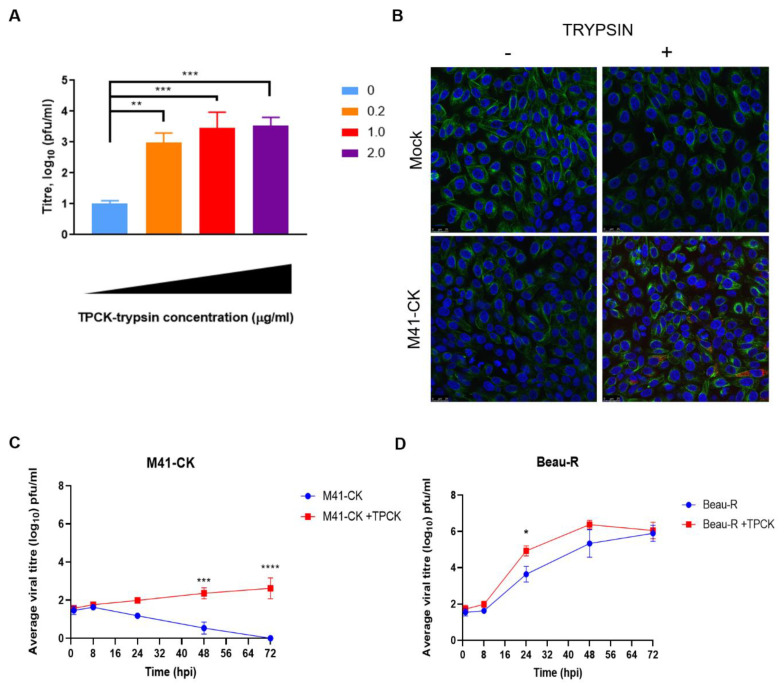
Trypsin enhances infectious bronchitis virus (IBV) replication in Vero cells. (**A**) Vero cells were infected with M41-chicken kidney (CK) (MOI = 1) diluted in 1XBES medium containing increasing concentrations of trypsin treated with l-(tosylamido-2-phenyl) ethyl chloromethyl ketone (TPCK-treated trypsin) from 0–2.0 µg/mL. Supernatant was harvested at 24 hpi and titrated on CK cells in triplicate. Data are representative of three biological replicates. Data were analysed by One-Way ANOVA followed by Tukey’s test for multiple comparisons and statistical differences from the untreated (0 µg/mL TPCK) values are indicated. ** indicates *p* < 0.01, *** indicates *p* < 0.001, **** indicates *p <* 0.0001. (**B**) Confocal images of Vero cells infected with M41-CK in the presence of trypsin (1.0 µg/mL) compared to mock infected and untreated cells. Cells were stained with monoclonal antibodies against dsRNA (Red) and α-tubulin (Green). Nuclei were stained with DAPI (Blue). White scale bars indicate 25µm. Replication kinetics of M41-CK (**C**) and Beau-R (**D**) in the presence of trypsin (1.0 µg/mL) were assessed in Vero cells. Cells were infected with IBV at MOI of 0.01. Supernatant was harvested at 1, 12, 24, 48 and 72 hpi and titrated on CK cells in triplicate. Average viral titres of at least three biological replicates are displayed with SEM. Statistical differences were calculated by Two-Way ANOVA comparing IBV strains and treatments.

To assess whether IBV was susceptible to trypsin treatment, the replication of the M41-CK strain was investigated in Vero cells in the presence of increasing concentrations of TPCK-treated trypsin. M41-CK is a laboratory-adapted IBV strain that exhibits a pathogenic phenotype in vivo and belongs to the Massachusetts (Mass) serotype that has been used extensively in vaccine research [9,47]. M41-CK displays a restricted tropism in vitro and is only able to be propagated in ovo, in ex vivo tracheal organ cultures (TOCs) and in primary CK cells; it is not able to replicate in Vero or DF-1 cells [19]. A defined quantity of M41-CK, 10,000 plaque forming units (PFU), in cell culture medium containing defined quantities of TPCK-treated trypsin was used to infect Vero cells. After the primary incubation the inoculum was replaced with cell culture media containing the same defined quantity of TPCK-treated trypsin used for the infection. An M41-CK with no trypsin control (referred to as untreated from this point) was included in the experiment. The quantity of infectious progeny virus at 48 h post-infection (hpi) was assessed by titration in CK cells (Figure 1A). In the absence of trypsin, very low titres of M41-CK were present in the harvested supernatant (<10^1^ PFU/mL). The addition of trypsin increased the titres significantly, with titres reaching >10^3^ PFU/mL when 1 or 2 µg/mL of TPCK-treated trypsin was added. At the lowest concentration of TPCK-treated trypsin, 0.2 µg/mL, the titre of M41-CK was also significantly increased compared to that produced in the absence of TPCK-treated trypsin (*p* < 0.005). To further investigate these initial results, which indicated the addition of TPCK-treated trypsin could enable M41-CK to replicate in a non-permissive cell line, immunofluorescence microscopy was used to confirm the presence of double stranded RNA (dsRNA) (Figure 1B). The level of dsRNA, a well-established marker of IBV infection [48,49,50], was visibly increased in trypsin-treated M41-CK infected cells compared to the non TPCK-treated trypsin M41-CK infected control. This further indicated that the addition of TPCK-treated trypsin enabled M41-CK replication in Vero cells. 

Following the results of the initial infections (Figure 1A,B), the replication kinetics of two IBV strains were analysed in the presence and absence of TPCK-treated trypsin. As well as M41-CK, the rIBV Beau-R was included in the assay to establish whether the presence of trypsin affected a strain that was already able to replicate in continuous cell culture. Vero cells were infected with M41-CK and Beau-R at a low MOI of 0.01. Viruses were diluted in cell culture media containing 1 µg/mL TPCK-treated trypsin and, after the primary incubation, this was replaced with cell culture media containing the same concentration of TPCK-treated trypsin. An infected, no-trypsin control (untreated) was included for each strain. Supernatant was harvested at 1, 12, 24, 48 and 72 hpi and titres of infectious viral progeny were assessed by plaque assay in CK cells (Figure 1C,D). In the absence of trypsin, the titres of M41-CK decreased over the duration of the experiment, with no detectable virus observed at 72 hpi (Figure 1C). With the addition of trypsin, however, M41-CK titres increased steadily over the 72-h time period, reaching a peak titre of almost 10^3^ pfu/mL at 72 hpi. Although the titres produced at 48 and 72 hpi during M41-CK TPCK-treated trypsin infection were relatively low when compared to Beau-R (Figure 1D), they were significantly increased in comparison the untreated M41-CK control sample (*p* < 0.0005), suggesting that the presence of trypsin had enhanced replication. Interestingly, although Beau-R is able to replicate in Vero cells in the absence of TPCK-treated trypsin (Figure 1D), there was a significant increase in viral titre at 24 hpi in the trypsin-treated samples compared to the untreated control (Figure 1D, *p* < 0.05). This difference, however, was not maintained at the later time points, with no statistical differences observed at 48 or 72 hpi. For both Beau-R and M41-CK, the addition of exogenous trypsin during infection in Vero cells resulted in increased yields of both IBV strains, albeit at different time points during the infection. This indicates that the replication of both strains can be enhanced by treatment with exogenous trypsin and, for M41-CK, the addition of trypsin facilitated entry into a previously non-permissive cell line. 

### 3.2. The Addition of Exogenous Trypsin Allows Sustained Replication of IBV M41-CK in Vero Cells but Not 4/91 

**Figure 2:**
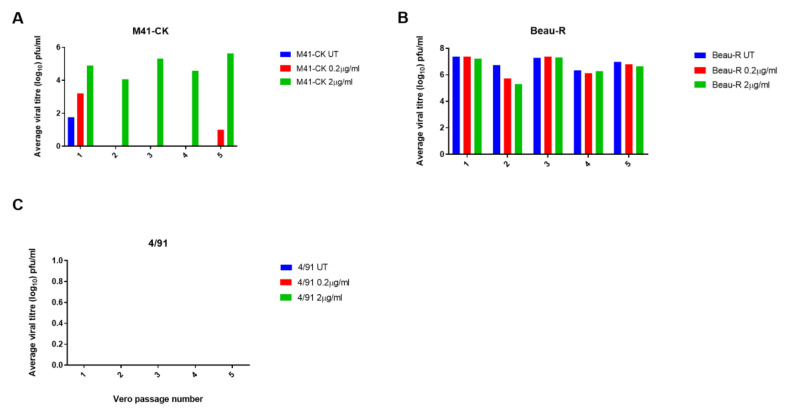
IBV replication in Vero cells is maintained over passage with trypsin in a strain-dependent manner. Three strains of IBV were passaged in Vero cells five times in the presence of TPCK-treated trypsin at a high (2.0 µg/mL) and low (0.2 µg/mL) concentration alongside untreated (UT) samples of each strain. Cells were infected with each IBV diluted at a ratio of 1:1 in serum free BES media containing each concentration of TPCK-treated trypsin. Supernatant was harvested at 48 hpi and used for the subsequent passage following the same protocol. Supernatant from each passage was titrated on CK cells in triplicate and viral titres were calculated (pfu/mL). The average viral titre at each passage is shown for M41-CK (**A**), Beau-R (**B**) and 4/91 (**C**).

Three strains of IBV, each exhibiting a different in vitro tropism, were passaged in Vero cells to assess if the increases in viral replication could be maintained and to monitor whether the viruses would adapt to trypsin. Alongside M41-CK and rIBV Beau-R, the classical respiratory strain 4/91(UK), which is of particular economic relevance in the UK [51], was included in these assessments. Notably, 4/91(UK) is a different serotype to both M41-CK and Beau-R (GenBank accession numbers JN192154, MK728875.1 and AJ311317 respectively) and exhibits a 17.6% and 17.7% amino acid sequence difference in the S glycoprotein in comparison to M41-CK and Beau-R, respectively. Propagation of both M41-CK and 4/91(UK) in cell culture would be highly beneficial for IBV vaccine research, as their in vitro tropisms are limited to ex vivo TOCs, embryonated eggs and additionally primary CK cells for M41-CK, but not 4/91(UK) [11]. Viral titres of M41-CK and Beau-R stocks were determined by plaque assay in CK cells prior to infection and the titre of the 4/91(UK) stock was determined in TOCs. The initial titres of each stock virus used for the first passage were comparable. Each virus was blindly passaged five times in Vero cells in the presence of TPCK-treated trypsin and titres at each passage were determined by plaque assay in CK cells in triplicate; an untreated control for each strain was also included. Virus presence in Vero cell supernatant was confirmed by PCR, using universal primer sets targeting the IBV 3′ UTR [52]. These universal primers allow for detection of a range of IBV strains and distinction between different strains, based on the size of the amplified PCR product. Using results from the dose-response experiment described in Figure 1A, two concentrations of trypsin were selected: a low concentration of 0.2 µg/mL and a higher concentration of 2 µg/mL. For M41-CK, titres of >10^4^ PFU/mL were maintained over five passages at the higher concentration of trypsin (Figure 2A). Despite a titre of 10^3^ PFU/mL at passage 1 (P1-Vero) no infectious virus was detected in the low concentration sample (0.2 µg/mL) at any other passage except at passage 5, in which a small quantity of M41-CK was detected. No PCR product could be generated from the supernatant using universal primer sets, indicating that the amount of virus present was negligible and fell below the limit of detection. The titres of Beau-R were largely unaffected at either concentration of trypsin, maintaining titres of >10^5^ PFU/mL, although there was a small reduction observed at passage 2 in comparison to the untreated control group, possibly due to increased cytopathic effect and a subsequent fall in virus production from the remaining viable cells (Figure 2B). In contrast to the other strains, 4/91(UK) was not detectable by plaque assay regardless of the trypsin concentration or passage number (Figure 2C). This could be attributed to the fact that 4/91 is unable to replicate in CK cells and thus would be undetectable by plaque assay [11]. To determine if 4/91(UK) was present, the supernatant was analysed by RT-PCR using universal primers targeting the 3′ UTR. At each passage, no 4/91(UK)-derived PCR product could be detected, indicating that the addition of trypsin had not enabled 4/91(UK) to replicate in Vero cells. For M41-CK and Beau-R, all samples containing virus detectable by plaque assay were also positive for IBV by RT-PCR. Taken together, these results show that passaging IBV in the presence of trypsin allows M41-CK replication to be maintained over five passages in Vero cells, but not 4/91(UK), indicating that the effect of the addition of exogenous trypsin is dependent on the IBV strain.

### 3.3. Additional S Protein Cleavage Products Were Not Detected Following Passage in the Presence of Trypsin

**Figure 3:**
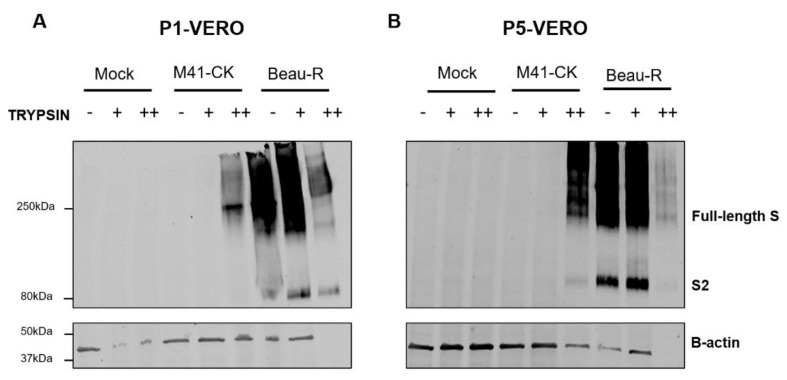
IBV spike (S) expression in the presence of trypsin. Cell lysates from mock, M41-CK infected and Beau-R infected Vero cells at P1 (**A**) and P5-Vero (**B**) were resolved by SDS-PAGE and analysed by Western blot. IBV S was detected using mAb 26.1 against the S2 subunit. Beta-actin was used as a loading control. Band sizes were compared with BioRad Dual Colour Standards. No change was observed in the size of the protein over the five passages. - indicates untreated/trypsin-free samples, + indicates samples propagated in BES medium containing 0.2 µg/mL TPCK-treated trypsin and ++ indicates samples propagated in BES medium containing 2 µg/mL TPCK-treated trypsin.

It was hypothesised that treatment with exogenous trypsin would result in additional cleavage of the IBV S protein, resulting in extra cleavage products that may be detectable by Western blot. To explore this, Western blot analysis of the S proteins expressed during infection in the presence and absence of trypsin was performed using a monoclonal antibody detecting the S2 subunit. S protein expression was monitored over the five passages in Vero cells (described in Figure 2) to further verify successful infection and identify any potential differences in cleavage products observed in the Western blots. Cell lysates were harvested from passage 1 and passage 5 (Figure 2A,B), separated by SDS-PAGE and probed using anti-S2 and anti-β-actin antibodies (Figure 3). After the first passage in Vero cells, the S glycoprotein from M41-CK was detected by Western blot only in the sample containing the higher concentration of TPCK-treated trypsin. The expression was not as strong when compared with the Beau-R samples, as the S2 band was not visible in the M41-CK derived samples (Figure 3A), indicating a smaller amount of S2 in this sample. In the M41-CK trypsin-treated samples harvested at passage 5, however, there is a clear band at approximately 80 kDa, corresponding to the predicted size of the S2 subunit (Figure 3B), indicating a greater amount of S2 compared to passage 1. In both the passage 1 and passage 5 samples, the bands relating to the Beau-R S glycoprotein produced in the higher concentration of trypsin are fainter when compared to the lower concentration. This is likely due to increased level of cytopathic effect (cpe) as a result of infection with the cell-adapted Beau-R strain, resulting in fewer remaining cells processed for Western blot analysis at 48 hpi. This is reflected in the quality of the β-actin staining observed in the higher concentration of trypsin, in which there was no visible staining for β-actin. Despite the lower level of Beau-R S glycoprotein detected in the higher concentration of trypsin, the Western blot analysis of both M41-CK and Beau-R indicates that the S protein is expressed during infection when exogenous trypsin is applied. The analysis also shows that no additional cleavage products were detected in any of the samples. 

### 3.4. Trypsin Treatment Leads to the Development of Trypsin-Independent Mutants

**Figure 4:**
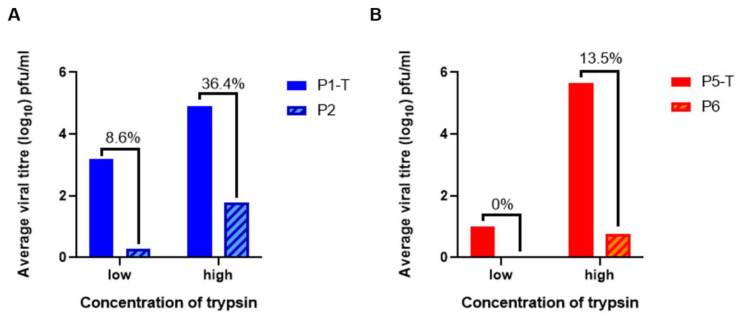
Passage in the presence of trypsin generates trypsin independent M41-CK mutants. (**A**) Supernatant from the first passage of M41-CK in Vero cells in a low (0.2 µg/mL) or a high (2 µg/mL) concentration of TPCK-treated trypsin (P1-T) was used to infect Vero cells in the absence of trypsin (P2). The quantity of viral progeny in each sample of supernatant was assessed by titration in CK cells in triplicate. Average viral titres from the two infections were directly compared in order to identify populations of trypsin-independent mutants. The percentage of trypsin-independent viruses present in the P1-T sample was then calculated. (**B**) Supernatant from the fifth passage of M41-CK in Vero cells in a low (0.2 µg/mL) or a high (2 µg/mL) concentration of TPCK-treated trypsin (P5-T) was used to infect Vero cells in the absence of trypsin (P6). Average viral titres from the two infections were directly compared to identify populations of trypsin-independent mutants. The percentage of trypsin-independent viruses present in the P5-T sample was then calculated.

It has been reported that passaging in the presence of exogenous proteases generates trypsin-independent mutants of PEDV, wherein the passaged viruses are able to re-infect Vero cells in trypsin-negative cell culture medium, displaying an adaptation to cell culture [40,53]. To investigate whether the same was true for the passaged M41-CK samples, supernatant from an early passage (P1-Vero) or the final passage (P5-Vero) in Vero cells (Figure 2A,B, respectively) was used to infect Vero cells in the absence of trypsin. The supernatant from these infections was assessed for viral progeny by plaque assay in CK cells. The titres obtained in the latter assessment were compared directly with the titre of the sample from the previous passage in the presence of trypsin, in order to calculate the proportion of viruses within the sample that were trypsin-independent (i.e., able to infect Vero cells in trypsin-free conditions, Figure 4). 

Figure 4A shows the comparison between the titre of virus obtained from the first passage of in Vero cells (P1-T) with the titre obtained when supernatant from these infections was used to infect Vero cells in the absence of trypsin (P2). In the low concentration of trypsin (0.2 µg/mL), the titre of M41-CK obtained from P1-T was >10^3^ PFU/mL. When the supernatant from P1-T was used to infect Vero cells in trypsin-free conditions (P2), a titre of <10^1^ was obtained, as measured by plaque assay on CK cells. This indicates that the majority of the population of viruses within the P1-T supernatant were trypsin-dependent. A small proportion of the viruses in the P1-T supernatant were able to infect Vero cells in P2, equating to 8.6% of the original titre. This may indicate the presence of trypsin-independent mutants in these samples. In the higher concentration of trypsin (2 µg/mL), the titre of the P1-T sample was close to 10^5^ PFU/mL. At P2, in the absence of trypsin, the titre obtained was <10^2^ PFU/mL. This indicates that 36.4% of the viruses in the P1-T sample, propagated in the higher concentration of trypsin, were able to infect Vero cells in the absence of trypsin at P2. 

These analyses were repeated after five passages of M41-CK in Vero cells in the presence of a low and high concentration of trypsin (P5-T). The supernatant from P5-T was titrated by plaque assay in CK cells, as previously described, and subsequently used to infect Vero cells in the absence of trypsin (P6). The supernatant from P6 was then titrated on CK cells and the titres were compared to analyse the population of potential trypsin-independent mutants (Figure 4B). In the lower concentration of trypsin, the titre of the P5-T sample was approximately 10^1^ PFU/mL. No virus was detected by PCR or plaque assay at P6, indicating that none of the viruses present in the sample were trypsin independent, or that the titre fell below the limit of detection. In the higher concentration of trypsin, at P5-T, the titre of the sample was >10^5^ PFU/mL. The titre of the supernatant from P6 was <10^1^ PFU/mL, equating to approximately 13.5% of the original P5-T titre. While this is a lower proportion than observed in the P1-T sample, it is important to note that there was a small population of viruses in the untreated sample that were able to infect Vero cells, but these were lost during the passaging process. This population may have contributed to the percentage of trypsin-independent mutants present at P2 in the absence of trypsin, but could not sustain replication to P5-T. 

Taken together these results indicate that trypsin-independent mutants may have been generated during the passaging process in Vero cells. They also indicate that the higher concentration of trypsin was more effective in generating these M41-CK mutants.

### 3.5. Trypsin Increases M41-CK and Beau-R Replication in Avian DF-1 Cells 

**Figure 5:**
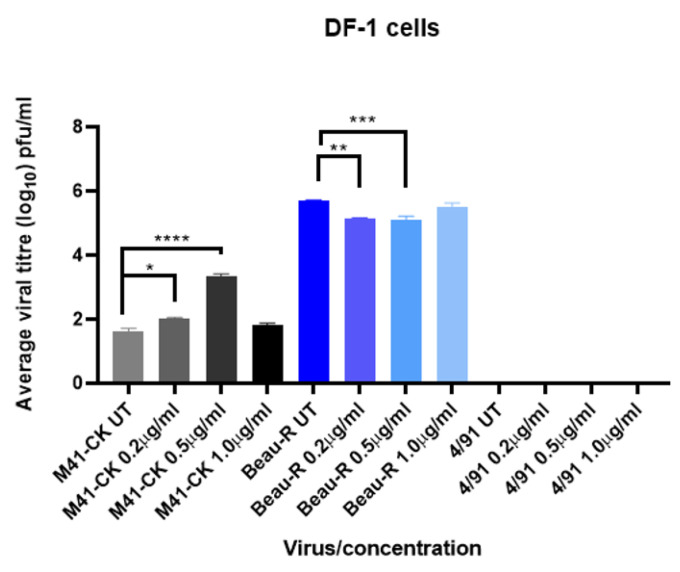
IBV replication in DF-1 cells is affected by trypsin. DF-1 cells were infected with IBV strains M41-CK, Beau-R or 4/91 (MOI = 0.1) diluted in BES medium either untreated (UT) or containing TPCK-treated trypsin at concentrations 0.2, 0.5 and 1.0 µg/mL. Supernatant was harvested at 24 hpi and titrated on CK cells in triplicate. Average viral titres are displayed with SEM. Statistical differences were calculated by One-Way ANOVA followed by Tukey’s multiple comparison test and are indicated for each strain. * indicates *p* < 0.02, ** indicates *p <* 0.01, *** indicates *p <* 0.001 and **** indicates *p* < 0.0001.

To assess if the effects of trypsin were cell type specific, IBV infections were repeated in DF-1 cells, a chicken fibroblast cell line originating from White Leghorn chickens [42]. The same IBV strains were investigated to assess how infection was affected with varying concentrations of TPCK-treated trypsin (Figure 5). Anecdotal evidence had suggested that these cells may be more sensitive to the action of trypsin than Vero cells, and so three concentrations, 0.2 µg/mL, 0.5 µg/mL and 1 µg/mL, were analysed. Cells were infected at a MOI of 0.1, in cell culture medium containing a defined concentration of trypsin. Following the primary incubation, the inoculum was replaced with cell culture medium containing the same defined concentration of TPCK-treated trypsin. An infected, no trypsin control was also included for each strain. Supernatant was harvested at 48 hpi, titrated by plaque assay on CK cells to quantify viral progeny and analysed by RT-PCR using universal primer sets as previously described. The effect of trypsin treatment in these cells was strain-dependent, similar to that observed in Vero cells (Figure 2). For M41-CK, significant increases in titres were identified at both the lower (0.2 µg/mL) and the intermediate concentration of trypsin (0.5 µg/mL) when compared to the untreated sample (*p* < 0.02 and 0.0001, respectively). There was however no difference in the titres obtained from higher concentration of trypsin (1 µg/mL) in comparison to the untreated sample, indicating that 0.5 µg/mL was the optimal concentration for M41-CK propagation in Vero cells. This could be due to increased trypsinisation of the cells at the higher concentration of trypsin, leaving fewer viable cells in which a productive M41-CK infection could be established. Beau-R replication decreased significantly in the presence of trypsin at the two lower concentrations (*p* < 0.001) but unlike M41-CK, was unchanged when propagated in the higher concentration. Although statistically significant, the reductions in titre observed at 0.2 and 0.5 µg/mL were only slight, indicating that the observed effect is not altogether biologically relevant, and that trypsin had little effect on Beau-R infection in DF-1 cells. As observed in Vero cells, 4/91(UK) was unaffected by the addition of trypsin. As 4/91(UK) is also unable to replicate in CK cells, this was confirmed by PCR, with no viral genome detected using universal primer sets. These results demonstrate that the effects observed for each strain are not dependent on cell type and further prove that trypsin can be used as a tool to facilitate IBV replication in continuous cell culture. Taken together, the results from the Vero and DF-1 cell infections (Figure 2 and Figure 5, respectively) show that the effects of exogenous trypsin on the productive replication of both M41-CK and Beau-R are not dependent on cell type. 

### 3.6. Trypsin Treatment Does Not Affect IBV Replication in CK Cells 

**Figure 6:**
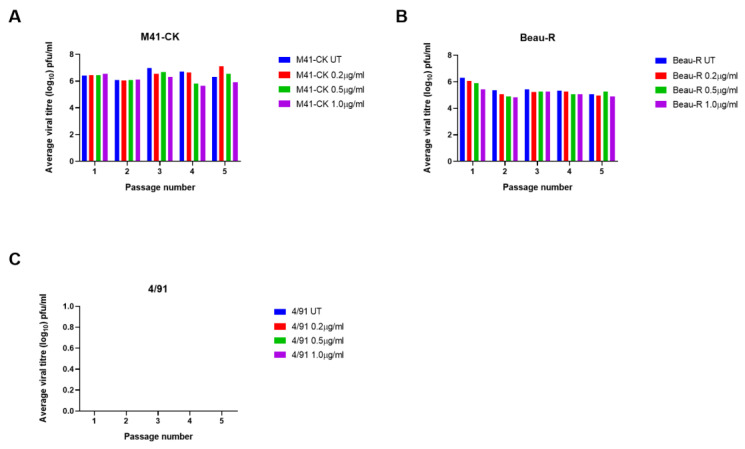
IBV replication is not affected by trypsin cleavage in CK cells. Three strains of IBV were passaged in the presence of TPCK-treated trypsin at three concentrations, 0.2, 0.5 and 1.0 µg/mL, alongside untreated (UT) samples of each strain. Cells were infected with each IBV diluted at a ratio of 1:1 in serum free BES media containing each concentration of TPCK-treated trypsin. Supernatant was harvested at 48 hpi and used for the subsequent passage following the same protocol. Supernatant from each passage was titrated on CK cells in triplicate and viral titres were calculated (pfu/mL). The average viral titre at each passage is shown for M41-CK (**A**), Beau-R (**B**) and 4/91 (**C**).

In parallel to the assessments in continuous cell lines, each IBV was passaged in CK cells at three concentrations, 0.2 µg/mL, 0.5 µg/mL and 1µg/mL (Figure 6). As noted for DF-1 cells, it was suspected that CK cells would be less tolerant of trypsin treatment and so a range of lower concentrations were assessed. An untreated control was also included for each strain. The primary objective here was to assess the effects of trypsin in a primary cell culture. CK cells are susceptible to infection by M41-CK and Beau-R but cannot be infected by the field strain 4/91(UK) [11]. For Beau-R and M41-CK, the addition of trypsin did not affect replication over the five passages. There was a small amount of variation in the M41-CK titres, but, overall, no change was observed (Figure 6A). Beau-R titres decreased slightly after the first passage in all treatment conditions but remained stable in all samples from P2 to P5-Vero (Figure 6B). The replication of the 4/91(UK) strain was unaffected by the addition of trypsin and no virus was detected at any stage in the passaging by either plaque assay or RT-PCR, mirroring both the results from the Vero and DF-1 passaging (Figure 2 and Figure 5). This further suggests that 4/91(UK) replication, unlike M41-CK infection, cannot be recovered in non-permissive cell types using exogenous protease treatment.

### 3.7. Passaging in the Presence of Trypsin in Vero Cells Results in Two Mutations within the M41-CK Spike Glycoprotein 

Viral RNA was extracted from the supernatant of the first (P1) and last (P5) passage in Vero cells (Figure 2), after which cDNA was generated for use in PCR analysis. PCR products were generated using primers which amplify the S gene for M41-CK passaged in the higher concentration of trypsin (2 µg/mL) and for all Beau-R samples. PCR products were Sanger sequenced and aligned with the published M41-CK and Beau-CK sequences (GenBank accession numbers MK728875.1 and AJ311317, respectively) and the sequences of the stock viruses used for the first passages to identify any mutations in the S proteins. In the M41-CK P5-Vero sample (2.0 µg/mL), two coding mutations were identified in the S protein sequence (Table 1). The first mutation was a nucleotide change from G to T at genome position 21689, resulting in an amino acid change from Arginine (R) to Isoleucine (I), at amino acid position 435 (R435I) in the S protein sequence. R435I is located in the C-terminal domain (CTD) of the S1 subunit (S1-CTD) and is close to the S1/S2 cleavage site at position 535. The second mutation from A to T at genome position 22156 resulted in an amino acid change from Threonine (T) to Serine (S), T595S. This position is located in the S2 subunit between the primary cleavage site (RRFRR) at position 535 and the putative location of the M41 fusion peptide [14]. Although both mutations are coding, no structural changes were observed (modelled using PyMOL and compared to the M41 S protein structure published by Shang et al. in 2018 with 100% confidence). For Beau-R, one mutation was identified in the S1 subunit of the glycoprotein, in the passage 5 sample from the higher concentration of trypsin. A nucleotide change in T20522A caused an amino acid change from Valine (V) to Aspartic acid (D) at position 50. This lies within the range of amino acids previously identified as critical for M41 binding [54].

Over the course of five passages in CK cells, M41-CK acquired a mutation at amino acid (aa) position 617 in the S2 subunit, inducing an N to L amino acid change (N617L). This mutation is present in all four samples, including the untreated sample, suggesting that the mutation was not induced by trypsin. No mutations were observed in the Beau-R S protein over the course of the passaging in CK cells. Notably, a mutation at aa position 617 was previously associated with the ability to replicate in Vero cells for Beau-R [21]. The mutation described here for M41-CK is not identical, but this indicates that this position is prone to mutation during cell culture adaptation. 

In summary, the results of the passaging experiments completed in Vero cells (Figure 2) and the DF-1 cell infections (Figure 5) demonstrated that M41-CK replication can be increased following the addition of exogenous trypsin in Vero and DF-1 cells. This effect of the addition of exogenous trypsin was, however, not reflected during the infection of CK cells (Figure 6). Exogenous trypsin treatment had no effect on 4/91(UK) replication in any of the cell types investigated, therefore indicating that the effect of exogenous trypsin treatment is dependent on the IBV strain.

## 4. Discussion

The addition of exogenous proteases, including trypsin, has been shown to allow the propagation of viruses including influenza, PEDV and MERS-CoV in vitro [37,38,39,40]. In this study, we demonstrate that the in vitro tropism of IBV can also be expanded using the addition of exogenous trypsin; however, the observed effect was both dependent on the IBV strain and the cell type. Whilst the titres of the M41-CK strain propagated in the presence of trypsin in the continuous cells lines Vero and DF-1 were significantly increased, the titres of the 4/91(UK) strain were not improved (Figure 2C, Figure 5 and Figure 6C). Unlike M41-CK, the 4/91(UK) strain was unable to establish infection in Vero, DF-1 or CK cells at any of the concentrations of TPCK-treated trypsin investigated. There are a number of amino acid differences between the 4/91(UK) S glycoprotein sequence compared to the M41-CK sequence, the majority of which are present in the S1 subunit containing the putative receptor binding domain (RBD). It is therefore possible that the 4/91(UK) strain is incompatible with receptors or attachment factors that are expressed on Vero, DF-1 or CK cells. This, in turn, would indicate that M41-CK and 4/91(UK) utilise different cellular receptors and/or attachment factors. While the primary receptor for IBV is yet to be elucidated, there is evidence of variation between the cellular proteins and co-factors used by different strains to enter cells [55,56,57]. This could explain why 4/91(UK) was unable to enter cell cultures, regardless of the presence of trypsin, if the virus and cellular receptors are incompatible. It has also been suggested that the restriction of coronavirus entry lies not only with the primary cell receptor but also with the compatibility of the viral proteins with the endogenous cellular proteases [37]. This theory is grounded in other research investigating the protease make-up of various cell types [31,58]. Another hypothesis is that the 4/91(UK) S glycoprotein may be resistant to cleavage by trypsin, due to a lack of accessible cleavage sites. Through analysis of the protein sequences of the Beau-R, M41-CK and 4/91 spikes using the online tool ExPasy Peptide Cutter, predicted trypsin cleavage sites were identified. In Beau-R, 78 sites were identified, compared with 76 in M41-CK and 74 in 4/91. A notable difference is a run of three R residues at aa position 687 in the M41-CK spike protein, which is not present in the 4/91 sequence. This is very close in position to the S2′ site and surrounding amino acids present in the Beau-R spike protein sequence at aa position 690 (RRKR), which has been identified as a determinant of cell tropism [21]. This could be the reason for the difference in the effects of trypsin on the different strains as this site in M41-CK may be susceptible to trypsin cleavage, but in a less efficient or stable manner than the cleavage of the S2′ site in Beau-R. As this is purely an analysis of protein sequence, these sites are predicted and not confirmed; thus, further investigation would be required to explore this hypothesis. Alternatively, the limiting factor for 4/91(UK) may be the replicase gene, rather than the S gene, negating any potential action of trypsin on the S protein. Future studies including strains from a range of different serotypes which display different in vivo tropisms to the strains described here would be essential to fully explore these strain-specific differences.

Unlike 4/91(UK), the replication of M41-CK in Vero cells was enhanced by the addition of exogenous trypsin (Figure 1C). Over five passages in Vero cells in the presence of trypsin, the titres of M41-CK increased (Figure 2A). Furthermore, M41-CK S proteins were only detected by Western blot following propagation in trypsin (Figure 3A). At P5-Vero, mutations were identified in the M41-CK S protein sequence, close to the S1/S2 furin cleavage site. Further investigations are required to ascertain if the mutations are stable and if they would be sustained over subsequent passages in the presence or absence of trypsin. Notably, a mutation arose in the S1 subunit of the protein, which is considered the main antigenic target during IBV infection [26]. S1 is also the most variable region of the S protein and mutations in this subunit can alter viral antigenicity [59,60]. The other mutation identified is located in the N-terminal region of the S2 subunit, which harbours the fusion peptide. This finding is consistent with results from PEDV experiments, in which the region of the S protein responsible for trypsin dependence was mapped to the N-terminal half of S2 [40]. It remains to be established whether the antigenicity of the mutated M41-CK S protein has been changed as a result of the passaging. This was recently noted by Menachery et al. following MERS-CoV propagation in the presence of trypsin, where a therapeutic antibody failed to neutralise the trypsin-treated virus [37]. An alteration in antigenicity would be an important consideration if this technique were to be applied to studying viruses for vaccine development. 

Following passaging in the presence of trypsin, M41-CK was able to re-infect Vero cells in the absence of trypsin, indicating the presence of trypsin-independent mutants (Figure 4). However, the low titres obtained from these infections suggest that further passaging would be required to amplify the trypsin-independent populations. Trypsin-independent mutants have been identified in similar studies with PEDV [40], with their development attributed to a potential selection pressure exerted by culture in Vero cells, which have been reported to secrete molecules that inhibit trypsin in cell culture [61]. There may also have been a small number of viruses within the M41-CK population that were able to replicate in Vero cells at a low level, which were only able to maintain replication following trypsin treatment. This is evidenced by the low titres of M41-CK present in the first passage in Vero cells without trypsin (Figure 2A). The two mutations, R435I and T595S (Table 1), identified in the spike glycoprotein and discussed in the previous paragraph, indicate that M41-CK has adapted in the presence of trypsin; however, the predicted structural analysis of the protein structure showed no alterations to the conformation of the spike protein trimer. Further research is required to say with certainty that the ability of the trypsin-independent mutants to replicate in Vero cells without the addition of exogenous trypsin is the result of the acquisition of these two mutations. If confirmed, these mutations may present a defined route to generating IBVs capable of replicating in continuous cell culture. IBV tropism has been investigated before, with previous research investigating large gene replacements required to alter in vitro tropism [11,19,20,21,62]. More recently, in vitro tropism was successfully altered through a few mutations in the S2 subunit, indicating that a more targeted approach could be applied [21]. Using the data described in Table 1, these mutations could be introduced into the M41 genome by reverse genetics, to assess if the resulting viruses are capable of unassisted replication in continuous cell lines. 

Interestingly, the addition of exogenous trypsin also influenced the replication of Beau-R, a strain already able to replicate in DF-1, Vero and CK cells. An increase in viral titre was observed during the infection of Vero cells at 24 hpi in comparison to the untreated control (Figure 1C), but this increase was not maintained at later time points. This largely reflects the results described for PEDV, in which only the non-cell adapted strain benefited from the addition of trypsin at during the entry stage of replication, although cell-cell fusion was increased under these conditions [40]. As Beau-R is already adapted to replication in Vero cells, this strain does not require trypsin to achieve a successful infection but may benefit from it at certain time points. The virus titres obtained from the passaging experiments in both Vero and CK cells fluctuated (Figure 2B and Figure 6B). This may be the result of excessive cleavage in the S glycoprotein, although there was no evidence of altered cleavage in this protein detected by Western blot (Figure 3). The reductions in viral titres observed during passaging experiments may equally be linked to the extensive cytopathic effect (CPE) induced by Beau-R infection in combination with the CPE induced by trypsin. This is evident in the reduced β-actin levels detected by Western blot in cell lysates from trypsin-treated cells (Figure 4B). 

Further investigation is required to explore the specific stage of IBV replication affected by trypsin cleavage in more detail. This has been investigated for the *Alphacoronavirus* PEDV, with reports suggesting that trypsin cleavage affects receptor-bound spike proteins, more so than unbound spikes within the virion [63]. This indicates that cleavage by trypsin affects the primary stages of virus entry, facilitating membrane fusion and therefore increasing virus entry and replication. This study also highlights the importance of protein conformation. It is suggested that the virion-bound spike proteins adopt a conformation that protects them from cleavage by exogenous proteases, whereas the conformational changes induced by receptor binding may expose trypsin cleavage sites that were previously masked. Proteases have also been implicated in facilitating coronavirus release from infected cells [64]. Whether this is true for gammacoronaviruses such as IBV remains to be determined, but this highlights that entry is not the only step where trypsin may be required. 

The investigations described here are purely in vitro, and careful consideration would be required if the passaged viruses were to be used for in vivo studies. Attenuation has been reported for PEDV isolates passaged in protease-containing media [53], suggesting that cleavage is associated with pathogenicity. In the context of IBV, S protein cleavage has recently been investigated in relation to the in vivo tropism and pathogenicity of QX-like viruses, where the insertion of the S2′ cleavage site into a QX-like strain completely changed the tropism of the recombinant virus. The virus was capable of crossing the blood–brain barrier to cause encephalitis in infected birds, a novel clinical sign never before reported for any IBV strain [65]. This demonstrates the importance of studying cleavage patterns in these viruses, and the possible outcomes of altered tropism, either in vitro or in vivo, especially when this is considered in terms of vaccine development.

Coronaviruses are known to jump the species barrier to devastating effect, as demonstrated by the outbreaks of SARS-CoV and MERS-CoV in 2003 and 2012, respectively. More recently, the emergence of swine acute diarrhoea syndrome (SADS)-CoV was found to have originated from circulating viruses in local bat populations [66,67,68]. Aside from the obvious health implications of these outbreaks, the economic fallout is best evidenced by the ongoing SARS-CoV-2 outbreak, causing mass disruption to societies and economies across the world. The use of extracellular proteases to assist S protein cleavage enables us to study how these viruses can overcome host barriers and infect new species, as they gain the ability to enter new cell types. This can also elucidate the role of host cellular proteases in the entry and replication of coronaviruses, as well as facilitating the study of more fastidious field strains for the identification of novel strategies for vaccine development against economically important diseases, including infectious bronchitis. The finding that some IBV strains that do not replicate in continuous cell lines can be amplified by the simple addition of trypsin is important for enabling the study of these viruses without excessive use of embryonated eggs. This also has implications for more efficient vaccine production as well as the rapid characterisation of emerging coronaviruses in response to outbreaks. Increased virus yield in cell culture would be extremely beneficial for cell-based vaccine production methods. This has been investigated for influenza viruses in MDCK cells and could also be applied to IBV [69]. There are also many potential applications for the use of trypsin treatment in basic coronavirus research techniques.

## 5. Conclusions

In this study, we have described a platform for expanding the in vitro tropism of the *Gammacoronavirus* IBV. This builds on a growing body of research surrounding the cleavage of coronavirus spike proteins and the associated changes in virus tropism. The results indicate that the effects are strain specific and the expansion of tropism is not shared across all strains of IBV, suggesting a slightly more complex story regarding the barriers faced during coronavirus entry and replication. This work provides proof of principle that trypsin treatment can be applied to gammacoronaviruses and a potential platform for further investigations into the applications of this technique.

## Figures and Tables

**Table 1:** Mutations in the M41-CK and Beau-R S gene following passaging in the presence of trypsin.

Sample	Cell Type	Treatment (Trypsin Concentration)	Original Sequence	Mutation	aa Position within S Sequence
M41-CK	Vero	2 µg/mL	R	I	435
M41-CK	Vero	2 µg/mL	T	S	595
Beau-R	Vero	2 µg/mL	V	N	50
M41-CK	CK	UT	N	L	617
M41-CK	CK	0.2 µg/mL	N	L	617
M41-CK	CK	0.5 µg/mL	N	L	617
M41-CK	CK	1.0 µg/mL	N	L	617

Amino acids (aa) are numbered from the start codon of the S protein in each IBV strain.
